# Young children fail to generate an additive ratchet effect in an open-ended construction task

**DOI:** 10.1371/journal.pone.0197828

**Published:** 2018-06-18

**Authors:** Eva Reindl, Claudio Tennie

**Affiliations:** 1 School of Psychology, University of Birmingham, Birmingham, United Kingdom; 2 School of Psychology and Neuroscience, University of St Andrews, Fife, United Kingdom; 3 Department for Early Prehistory and Quaternary Ecology, University of Tübingen, Tübingen, Germany; University of Edinburgh, UNITED KINGDOM

## Abstract

The ratchet effect–the gradual accumulation of changes within a cultural trait beyond a level that individuals can achieve on their own–arguably rests on two key cognitive abilities: high-fidelity social learning and innovation. Researchers have started to simulate the ratchet effect in the laboratory to identify its underlying social learning mechanisms, but studies on the developmental origins of the ratchet effect remain sparse. We used the transmission chain method and a tower construction task that had previously been used with adults to investigate whether “generations” of children between 4 and 6 years were able to make a technological product that individual children could not yet achieve. 21 children in a baseline and 80 children in transmission chains (each consisting of 10 successive children) were asked to build something as tall as possible from plasticine and sticks. Children in the chains were presented with the constructions of the two preceding generations (endstate demonstration). Results showed that tower heights did not increase across the chains nor were they different from the height of baseline towers, demonstrating a lack of improvement in tower height. However, we found evidence for cultural lineages, i.e., construction styles: towers within chains were more similar to each other than to towers from different chains. Possible explanations for the findings and directions for future research are suggested.

## Introduction

The ability to produce cumulative culture–i.e., cultural traits (e.g., technological products, instrumental skills and knowledge) that could not have been created within a single lifetime but instead are the result of an evolutionary process over time and individuals (a phenomenon called the *ratchet effect* [1])–is often argued to be a uniquely human phenomenon and has recently even been marked as the “secret of our success” [2–6]. Trying to identify the reasons for this uniqueness, researchers have started to study the cognitive mechanisms underpinning cumulative cultural evolution [7–20]. The current literature suggests high-fidelity social transmission and a capacity for innovation as the main–but perhaps not the only–cognitive requirements for cumulative culture, alongside social and demographic factors which foster cumulative culture, such as group size and the interconnectivity of social networks [13,21–28], but see [29].

In order to study the role of social learning and innovation for the production of the ratchet effect, researchers use the transmission chain method as an experimental design to simulate cumulative cultural evolution under controlled laboratory conditions. In these studies, a series (“chain”) of participants are sequentially asked to take part in a task (e.g., building a weight-carrying device [19]). Participants in each position of the chain (apart from the first position) are presented with socially generated information, namely with the goal of the task, the actions, and/or final products (endstates) of participants in earlier positions of the chains (or even with teaching and communication). By varying the type of information presented as well as the combination of these types (e.g., actions, goals and endstates vs goals and endstates), the transmission chain paradigm allows to identify which social information (or which combination of different pieces of information [30]) is necessary for a ratchet effect to emerge.

Using such transmission chains, some studies suggested that action-copying (a component of imitation) is not always necessary for the ratchet effect to occur: Cultural products with a transparent physical structure could be reverse-engineered, and so endstate emulation was seemingly sufficient to produce a ratchet effect in these cases [9,20]. However, the relative ease with which adults produced a ratchet effect in these tasks (paper plane or basket making) could have been due to carry-over effects from life outside the laboratory: the participants likely possessed some retrievable previous cultural knowledge (*ontogenetic cultural intelligence* [2,31,32]) about how to succeed in the tasks and the endstate demonstrations could have helped to retrieve this knowledge successively along the transmission chains. Thus, ontogenetic cultural intelligence is a potential confound in these tasks, making it difficult to determine whether the ratchet effect is possible via endstate copying alone. It is possible that the ratchet effect still requires other social learning mechanisms to emerge, such as imitation [33] or, more generally, process copying rather than product copying [5]. One of the few methodological ways to minimize the influence of past learning is studying young children. That way, and by using novel or unusual tasks, ontogenetic cultural intelligence–while not fully excludable–is less likely to be a confounding variable. This approach was taken in the current study.

Researchers also used what are considered to be cognitively more opaque tasks such as making a virtual fishing net or knapping a stone tool and found that endstate emulation was *not* sufficient to produce or maintain cumulative culture [14,15,19]; however, the short trial length in Morgan et al. [15] might have suppressed such effects from emulation alone. In sum, transmission chain studies on human adults have shown that imitation and possibly also endstate emulation are faithful enough to support a ratchet effect (but see [29] for further critique of method details).

Young children already possess one crucial pre-requisite for the ratchet effect, the capacity for high-fidelity copying. From 14-months, infants flexibly switch between imitation and emulation [34]. This raises the question whether groups of children are also able to *produce* (rather than only copy) cultural traits that they could not have achieved individually or whether this ability is restricted to adults and what the underlying reasons could be.

We call these traits that individuals cannot acquire on their own but only via high-fidelity social learning *culture-dependent traits* (following [17] and [4]). Note that we explicitly do not phrase our question as whether children can produce “cumulative culture”–we prefer to use the broader term culture-dependent trait. We draw this distinction for the following reasons: Culture-dependent traits describe traits that a given individual or group of individuals can only acquire through faithful social learning; and so the definition comprises both cumulative culture (traits that no individual of the species could invent on his or her own during his or her lifetime) *and* other traits that are beyond the spontaneous reach of the individual(s) in focus (e.g., young children) but that are still within the spontaneous reach of *other* members of the species (e.g., adults). For example, it has been found that when 40 4-year-olds were asked to build something very tall, they did not spontaneously make a certain efficient tower structure: a tripod [17]. Yet, children were able to make a tripod when they received a demonstration from an adult. Thus, the tripod is a culture-dependent trait for 4-year-old children, but it might not represent cumulative culture for adults. For it might well be that the tripod could be invented by any independent individual at some later point in the lifespan without social learning (as a thought experiment think of a human raised on a desert island, [1]), thus violating the definition for cumulative culture. The notion of culture-dependent traits allows researchers to study the developmental origins of the ability to learn novel cultural traits without producing confusion about nomenclature: Cumulative culture is usually assumed to be created by *adult* members of a group who have acquired reasonably large and deep cultural intelligence. In contrast, children are thought to primarily learn about and absorb their cultures rather than to add to them [35].

Currently, we know little about the origins of the ratchet effect in humans, and so we aimed to explore the developmental roots of cumulative cultural learning. Studying the ratchet effect in children can help to delineate its necessary cognitive and motivational requirements because children have not yet gained as much cultural knowledge as adults and so the cognitive processes can be studied more (but of course not completely) independently from ontogenetic cultural intelligence. Children have only recently become a research focus in the field of cumulative cultural learning [12,18,36–40]. Initially, researchers identified a sub-form of the ratchet effect–the *subtractive ratchet effect* [18]: Flynn [36] showed that groups of 2- and 3-year-olds “weeded out” causally ineffective actions seeded by an experimenter when retrieving a reward from a puzzlebox. Using the same apparatus, McGuigan and Graham [40] showed a similar effect in 5-year-olds–but not in 3-year-olds, who instead copied both relevant and irrelevant actions. Tennie et al. [18] seeded chains of 4-year-olds with inefficient ways of carrying rice from one place to another and also found that ineffective solutions were dropped along the chains. These studies suggest that behavioural sequences in children can undergo an evolution from suboptimal initial actions towards more efficient ones. However, as the target behaviours in these studies were still within children’s spontaneous reach (as indicated by children’s baseline performance), these studies show that children are able to make modifications to behaviours which are still fully within the scope of individual invention. Tennie et al. [5] labelled this type of improvement, often directed towards a “gravitational center”, a “step-wise tradition”, as contrasted with actual culture-dependent behaviours that go beyond the inventive capacity of individuals.

However, it is the study of the *additive type* of ratchet effect [18], defined as the build-up of cultural improvements in trait complexity and or efficiency *beyond* a level that could be reached individually throughout a lifetime, leading to open-ended outcomes rather than to asymptotic optimizations [4], that promises insights into the developmental origins of human cumulative culture. Of course, even open-ended cumulative culture may eventually level off (e.g., the time requirements for learning traits may exceed the length of a human life, despite knowledge acquisition being facilitated by technological advances [41]), and so even open-ended cumulative culture eventually may become asymptotic. The real difference between the subtractive and the additive ratchet effect then is that the subtractive ratchet arrives at the asymptote through a “downward” process of reduction, whereas the additive ratchet effect does so via an “upward process” (i.e., channelling versus hill climbing). Note that there is now clear evidence for the subtractive ratchet effect in non-human animals [42,43], but the additive ratchet effect has (so far) been more elusive–except for postulated, untested cases (e.g., brush tip tools in chimpanzees [44] or wheat washing in Japanese macaques [45]).

Few studies so far have aimed to investigate the additive ratchet effect in children: Dean et al. [12] presented groups of 3- to 4-year-olds with a three-stage puzzlebox whose stages could be completed sequentially. The authors found that the majority of the groups had at least two children solving the third stage of the box and concluded that 3- and 4-year-olds were capable of “cumulative cultural learning” (p. 1117). However, despite the fact that the box technically allowed for an increase in the number of solved stages, it was unclear whether it also captured an increase in complexity or efficiency beyond a level that could be reached individually: a baseline condition controlling for such asocial invention was missing. And it is only the inclusion of such a baseline condition that will allow to identify whether the task at hand is a culture-dependent trait (see below for different methods on matching the baseline to the experimental condition). In contrast to [12], the rice-carrying study by Tennie et al. [18] did include a baseline condition. Yet, the authors found no evidence for chains of 4-year-olds generating efficient techniques of rice transport that would have gone beyond the individuals’ performance. However, the task was probably more suitable for inducing a subtractive rather than an additive ratchet effect as some of the more efficient solutions of the task–e.g., using the containers provided to carry as much rice as possible–proved to be well within children’s reach and any improvements along the chains were those that selected against demonstrated inefficient means, so that children in the transmission chains eventually converged to behaviour of baseline performance (i.e., using a certain sub-set of materials to transport the rice). Thus, this finding should also be regarded an example of a step-wise tradition.

Reindl et al. [17] investigated whether children already possess one of the two crucial requirements for producing cumulative culture–namely, the ability to copy culture-dependent traits (see [34,46,47] for studies who investigate this question in the social-conventional, rather than technological, domain). They adapted a task that had previously been used in transmission chains with adults–the spaghetti tower task [7]–but tested children individually, as they were solely interested in whether children could *copy*, rather than *produce* a culture-dependent trait. Four- to six-year-old children were assigned to one of three conditions–a baseline, an endstate demonstration, and an action-plus-endstate demonstration condition–and asked to build something from plasticine and sticks that was “as tall as possible”. Children in the two demonstration conditions were additionally presented with a culture-dependent trait: an efficient and stable tripod-shaped tower, which, crucially, children in the baseline condition never produced spontaneously. Children in the action-plus-endstate demonstration condition watched the experimenter build the tripod, those in the endstate demonstration condition were presented with the ready-made tripod but without any action information. Children in both demonstration conditions were able to build taller towers than those in the baseline and, crucially, some children in both conditions also spontaneously reproduced the tripod shape. Thus, this study demonstrated that the ability to *copy* a culture-dependent technological product–a key ingredient for the production of the ratchet effect–is already present in pre-schoolers.

Most recently, McGuigan et al. [39] employed an open-diffusion design (in which several participants are presented with a task simultaneously so that the number and nature of learning instances is left open) in order to study children’s cumulative cultural learning using a puzzlebox consisting of four levels. Each level contained rewards of increasing desirability, which could be extracted via four types of exit per level, and the solution of higher levels required different levels of actions (level 1: use of a finger, level 2: tool use, level 3: manufacture and use of a tool, level 4: manufacture and use of a more complex tool). The authors found evidence that groups of children solved the levels of the box in an ascending order: All of the nine open diffusion groups achieved their first solutions on level 1 (even though the solution of level 4, containing the most desirable reward, did not require solution of level 1), and solutions on higher levels always occurred only later in the experiment. It was also found that groups of children were able to reach stages that asocially tested control children did not reach on their own (L3 and L4). However, these results need to be interpreted with caution for several reasons. First, testing time for the asocial control children did not match that of the social learning condition. In general, matching a baseline to a transmission chain or open diffusion condition can be done in at least two major ways (see S1 Table): Method 1 is to fix the testing time for each individual across conditions to one standard time; for example, in the current study, the individual contribution of each child, regardless of condition, was limited to 10 min. The difference between conditions then lies in the *total* (accumulated) amount of time that has gone into the final product (in our study, the products at the end of the transmission chains are the result of 10 children building for 10 min, (thus 100 min total building time) compared to 10 min for each of the products in the baseline). Method 2 is to start with the overall testing time for each transmission chain (to use our example again, this would be 100 min) and match the baseline accordingly (e.g., to test each baseline child for 100 min, split into several testing sessions); this results in varying testing times per individual between conditions (see also the design used in [20,43]). While it is not clear whether either of these methods is more preferable, both methods effectively isolate a “social learning factor” that differentiates between the conditions: in Method 1, even though each individual always contributes 10 min, these 10 min differ in whether individuals have access to social information; in Method 2, the amount of time that goes into the final product is the same, but the difference lies in whether this time was provided by a single person learning asocially or several individuals learning socially.

Coming back to the McGuigan et al. [39] study, since this study used an open diffusion design, it was unfeasible to fixate the contribution of every individual in the open diffusion condition to a specific amount of time, thus ruling out the use of Method 1 (see S2 Table). The total amount of time their puzzlebox was presented to children in the open diffusion condition was 4 h, i.e., the box was available for all children to interact with for 4 h, but the individual interaction times were shorter, as not all children spent the entire time with the box. This overall time, however, was not matched in the baseline (as would have been expected by using Method 2): baseline children were maximally given 45 minutes overall (15 min initially, with an additional two sessions of 15 min). Note that the two later 15 min sessions were only granted to selected children, namely those baseline children who obtained a reward in the first session (thus leading to a reduction of the baseline sample size). In addition, another potential confound in the comparison between the group social learning condition and the baseline is that only baseline children were tested asocially, i.e., not only did the baseline children lack the opportunity for social learning, but they could not benefit from any social facilitation effects either [48–50]. In other words, it could have been possible that the mere presence of other children in the testing room–even if these children did not interact with the apparatus–could have, e.g., counteracted shyness and motivated the baseline children to interact more with the apparatus. It is noteworthy that despite the shortness of the asocial control condition (less than 20% of the time that what was granted to the group-tested children), the effective reduction of the sample size and the lack of social facilitation in the asocial control condition, McGuigan et al. [39] found that asocial control children were still able to reach level 2 of the puzzlebox on their own. This leaves open the possibility that in a control condition where children (crucially, *all* children, not just a subset) were given more time and were not deprived of social facilitation effects (e.g. by adding idle bystanders), children might have reached even higher levels on their own. In addition, even though the study consisted of additional control conditions with adjusted apparatuses, not all necessary control conditions were implemented (“L3-only” control was missing). Therefore, what can be safely concluded from this study is that asocially tested children were slower in solving levels 1 and 2, but it remains unclear whether reaching higher levels of the apparatus represents a culture-dependent trait for children. Finally, even though the choice of an open diffusion design allows to study children’s cumulative cultural learning in a potentially ecologically more valid context (because the design leaves open who may learn from whom), validity is traded against controllability. As the authors acknowledge, the interpretation of the data remains tentative as it consists only of correlational data. In sum, the authors’ conclusion that social learning of any kind was *required* to reach higher levels in their task is not shared in our reading of the evidence. Designs using tightly controlled social demonstration types are required to determine the absolute role and identity of the social learning mechanisms underlying cumulative cultural learning.

The current study investigated the ratchet effect in children 4.5 to 6 years of age using a tower construction task in a transmission chain design. In contrast to previous studies [12,18] our task was open-ended and allowed for an additive ratchet effect to occur. We randomly assigned children to one of two conditions: a baseline and a transmission chain condition. We fixed the testing time across conditions to be 10 min per individual (see above for “Method 1” of matching experimental and asocial conditions). Since the tower task is a relatively transparent task for both adults [11] and children [17], requiring no action information for participants to copy, the children in the transmission chain condition of the current study received an endstate demonstration: they were presented with the towers made by children in the two preceding generations. Children in both the baseline and the transmission chain condition were tested asocially–they had no contact to other children during the study. This choice of demonstration also rendered the implementation of a transmission chain study with children much more feasible. We chose to present children with two (rather than e.g., one or three) exemplars to be in alignment with the procedure of previous adult studies and to increase comparability with these [7,16,19,20].

We hypothesized that children in the transmission chains would be able to produce a ratchet effect, i.e., that they would build upon each other’s knowledge and gradually build taller constructions, so that the towers in the last position of the chains would be taller on average than constructions made by individual baseline children. We also expected that children in the transmission chains would be able to create novel, more efficient tower designs, such as the tripod. One might argue that one should *not* expect children in the chains to be able to make a tripod, given that Reindl et al. [17] found that the tripod is beyond the spontaneous ability of 4- to 6-year-olds and that children this age need a demonstration in order to build a tripod on their own. However, this strikes us as unlikely: While we do not expect individual children to come up with a culture-dependent trait on their own (by definition), we argue that there are two ways in which individuals can acquire culture-dependent traits: 1) by high-fidelity social learning of an already existing culture-dependent trait (e.g., via adult demonstration, as demonstrated by [17]) and 2) by a group of individuals pooling their knowledge and ideas, leading to a co-construction of a culture-dependent trait (ratcheting) [33], as we might expect from the transmission chain condition in the current study. In other words, while in the first case a culture-dependent product is acquired through high-fidelity social learning such as imitation, emulation or teaching via these mechanisms, the second way describes how culture-dependent products come into existence in the first place: As they are by definition beyond the innovative capability of any individual, culture-dependent traits can only emerge gradually through a ratcheting process of social learning and innovative efforts from a connected group of individuals. Even though in our endstate demonstration condition children were tested individually and so could not interact with the other participants in their chain, this condition still effectively connected the products of the children’s efforts. It granted the possibility for social learning via emulation: by emulating the constructions made by previously tested children, children can also add upon the observed ideas and designs, which can then be reflected in their own constructions etc., and so the children can–as a virtual group–gradually improve the design of their constructions across the chain. Children in later generations can thus begin their construction session at a “higher” starting point: They can copy or extract accumulated information from *previously* made towers and/or can learn which design does not work or what not to do [17,51]. And so, we expect that even in an endstate demonstration chain condition, in which participants are tested asocially, a culture-dependent product such as the tripod could gradually occur. Indeed, studies with adult participants have shown that when individuals are given the opportunity to share information in pairs or small groups, they outperform individual participants and also create products (e.g., virtual totems) that participants working alone could not have achieved [22,28]. However, even if children in the transmission chains would not be able to make a tripod, there would still be the possibility of finding a ratchet effect: Children were given the freedom of choice of a design and so children could have invented other culture-dependent designs. A second way to detect a ratchet effect is by measuring tower height, as was also done in adult studies [7]; i.e., by analysing whether tower height would gradually increase over the transmission chains, resulting in towers in the last positions of the chains exceeding the height of baseline towers.

## Materials and method

### Study design

Children were assigned to a baseline (*n* = 21) or a transmission chain condition (*n* = 80). In the baseline, children were asked to build something as tall as possible using plasticine and sticks, thus replicating the baseline condition in [17]. This condition was contrasted with children’s performance in the transmission chain condition. For this condition, we implemented eight chains with a length of ten children each (chains separated by gender: 4 boy chains, 4 girl chains). Both the number of chains per condition and the chain length we chose exceed what is usually implemented in transmission chain studies with children (see S3 Table). Children assigned to the transmission chain condition were allocated–in succession–to one position (1–10) within a single-gender chain. As in the baseline, children were tested individually and asked to build something as tall as possible. In addition, they had the opportunity for social learning as they were shown the final constructions made by their two immediate predecessors (by design, this only happened for children in positions 3 to 10; children in position 2 only saw the construction of the first child, and children in position 1 did not have access to any social information, experiencing the same situation as baseline children).

### Participants

The final sample consisted of 101 children (51 boys) between 4 years 5 months and 5 years 8 months (*M*_age_ = 57.87 months, *SD* = 3.40 months) tested in the UK (in a science museum (*n* = 56) and a zoo (*n* = 32) of a metropolitan area) and in Germany (in a school building outside of school hours (*n* = 9) and a nursery (*n* = 6)) of a rural area). Children were assigned either to one of the single-gender transmission chains (8 chains in total, 4 boy chains, 4 girl chains; *n* = 101) or a baseline condition (*n =* 21, 11 boys). The ethnic composition of the sample was 62.7% White British, 13.7% White German, 12.7% Asian British, 8.8% other Mixed, 1% other White. An additional 16 children were tested but had to be excluded from the study because they did not meet the required age range (*n* = 4), because they answered the control question wrong (*n* = 3), because of experimenter error (*n* = 2), and seven children had to be excluded because they were tested in a transmission chain following a child who did not meet the required age range and thus had to be excluded. In the final sample, children in the transmission chains (median = 58 months) were found to be slightly older than children in the baseline (median = 56 months, Mann-Whitney-U-Test: *U* = 562.5 *p* = .020), a point to which we return in the discussion. In the transmission chains, there was no correlation between age and generation, *r* (78) = .039, *p* = .733. Participants were recruited via information letters sent to schools and flyers handed out to parents at various events. Written informed consent was obtained by children’s parents or caregivers before testing started. Parents were present during testing for all children except for five children tested in the nursery. Children were rewarded with stickers, a certificate, and a voucher regardless of success. Ethical approval was granted by the University of Birmingham, UK, STEM Ethical Review Committee. The study was carried out in accordance with the approved guidelines.

### Material

We used the same commercially available materials as in [17] (30 white lollipop sticks, 150mm x 4.5mm; Newplast green plasticine) with the exception that we provided children with 100 g instead of 70 g of plasticine in order to not impose any arbitrary external limits to a potential accumulation in tower design. We used the timer function on an iPad to show children their time progress during the task (analog and digital display). In the transmission chain condition, we also used a movable table (70 x 42 x 18 cm) to present the constructions built by the preceding participant(s).

### Procedure

Children were tested individually by the same female experimenter (E). Both were sitting on the floor, at a low table (h = 15 cm). Opposite of the child, around 2 m away, there was an adult-sized table underneath which there was the moveable table of the same height as the low table the child was sitting at and which E placed the demonstration tower(s) on. A black tablecloth hanging from the big table prevented the view of the constructions at the start of the trial. After the warm-up game, children were asked a control question to see whether they understood the word “taller”: Children were shown two plastic giraffes that differed in height (“mother” and “calf”) and were simply asked to point to the one that was taller.

At the beginning of the construction task, children in both conditions were presented with the building material (100g of plasticine and 30 plastic sticks) and the goal of the task: to build something “as tall as possible”. The demonstration of the material was carried out as in [17]. In the transmission chain condition, participants were then presented with social information (apart from children in position 1). E said: “So before you start, let me show you what another child (other children) did earlier!” E lifted the tablecloth and moved the table presenting the construction(s) closer, so that it was ~ 100–120 cm away from the child. The presented tower(s) were mainly the original towers that had been built by previous participants tested earlier that day or the day before (63 out of 83 towers). However, since testing took place over several sessions spread out over several months, sometimes the end of a session did not coincide with the end of a chain, so that we had to disassemble children’s towers and reassemble them at the beginning of a new session. Out of 72 towers that were demonstrated to children (8 chains x 9 towers), we had to rebuild 18 from photos of the original towers.

E looked at the constructions for 5 sec, then said: “Now you try to build something that is very tall!” Children in both conditions had a maximum of 10 min to complete the tasks. In previous studies we experienced that some children were concerned that they would run out of time, which might have prevented them from concentrating fully on the task. Therefore, we displayed a timer visible to children throughout the task which allowed them to track their progress. E also explained that there was no need to rush as the game was not a race. Children were then encouraged to start building. Testing stopped when 10 min were over or when children refused to further engage in the task even after having been encouraged to continue several times. Most children used the full 10 min (67 out of 101 children); of the 34 children who did not use the full trial length, 28 children were building for 9 min or longer, one was building between 8 and 9 min, two between 7 and 8 min, another two between 6 and 7 min, and one for 5 min 40 sec. In the transmission chains, the towers of the one or two previously tested children were present during the entire building time (maximum 10 min).

### Coding and statistical analysis

For each child, we measured three variables (live-coded): *tower height*: the height of the tower that was standing on the table at the end of the trial; *tower shape*: the shape of the tower, using the classification system used in [17]; and whether or not children produced a *tripod* (yes or no), which is an efficient solution to the task and likely represents a culture-dependent trait for children between 4 and 6 years. With regard to tower shape, we grouped towers into several categories. First, we determined the tower height in sticks, i.e., we counted how many sticks were vertically combined on top of each other (“combining” meaning two sticks joined on top of each other with a piece of plasticine, while an overlap of up to half the length of a stick was allowed). With this classification, each construction could be assigned to one of the following groups: level-0-constructions (towers that were smaller than the height of one stick, e.g., towers consisting only of plasticine); level-1-constructions (constructions with one (or more) sticks placed vertically into a plasticine base); level-2-constructions (constructions in which two sticks were combined on top of each other); and–applying the same logic–level-3- and level-4-constructions. Within each category, we further grouped the constructions based on their shape (see Table 1).

**Table 1:** Shapes and heights in “stick levels” of the constructions made in the baseline and the transmission chains.

			Number of constructions	
Tower height in sticks	Tower shape	Shape description	Baseline	Transmission chains
Level 4	Level-4-2-leg-tower	Level-4-tower with 2 legs	-	1
	Level-4-tower	4 sticks combined vertically on top of each other (at least 1 stick per level)	-	1
Level 4 Total			-	2/80 (2.5%)
Level 3	Elevated level-3-tower	As level-3-tower, but with increased plasticine base	-	1
	Level-3-tower	3 sticks combined vertically on top of each other (at least 1 stick per level)	-	3
Level 3 Total			-	4/80 (5%)
Level 2	Modified Level-2-tripod	4 or more legs at the base, combined with piece of plasticine at the top, on top of this 1 or more vertical sticks	-	2
	Level-2-3-leg-tower	Level-2-tower with 3 legs arranged in a line	1	-
	Level-2-2-leg-tower	Level-2-tower with 2 legs	-	6
	Level-2-tower	2 sticks combined vertically on top of each other (at least 1 stick per level)	4	15
	Other level-2-constructions		1	3
Level 2 Total			6/21 (28.6%)	26/80 (32.5%)
Level 1	Broken level-3-tower	Level-3-tower that broke down to level-1-height	-	1
	Level-1-tower with 2 legs	2 sticks with plasticine base combined with plasticine at the top	-	1
	Level-1-tower with plasticine cap	As level-1-tower, but with pieces of plasticine on top of 1 or more sticks	-	1
	Elevated level-1-tower	As level-1-tower, but with increased plasticine base	2	
	Level-1-tower	At least 1 ball of plasticine with at least 1 vertical stick on top	2	14
	Hedgehog	Ball of plasticine from which several sticks protrude upward and/or to side	5	21
Level 1 Total			9/21 (42.8%)	38/80 (47.5%)
Level 0	Level-0-tower	Construction involving sticks and plasticine, smaller than height of 1 stick	-	3
	Plasticine-only tower	Construction made from plasticine only	5	2
	Fallen tower	Tower of any height that didn't stand on its own	1	5
Level 0 Total			6/21 (28.6%)	10/80 (12.5%)

The datasets generated and analyzed during the study are available as a Mendeley Dataset. We first analyzed the heights and shapes of the towers in the baseline and transmission chains. Since children in the baseline and those in positions 1 in the chains experienced the same test situation (no social information present), we expected to find no difference in tower height between these two groups (tested with an independent samples t-test). We then analysed the data with regard to three main questions:

#### Was there an increase in tower height across generations?

To investigate whether there was a significant improvement in tower height along the chains, we conducted three analyses: We examined whether the average tower height in position 10 across the chains was significantly different from the average height of the towers in position 1 using a Wilcoxon signed rank test. Following Caldwell & Millen [9], we did the same analysis comparing the average height across the last three towers (position 8, 9, 10) with the average height across the first three towers (position 1, 2, 3). Third, we used a new implementation of Page’s L test for R using the R package “cultevo” [52] to examine whether there was a monotonic increase in tower height along the chains. Note that Page’s test has been criticized as being too sensitive as even a single step-wise increase within the chain would yield a significant test result [48]. Therefore, researchers have started to use linear mixed models (LMM) with a maximum random effect structure as a more appropriate test (Caldwell & Atkinson, pers. comm.). Therefore, we confirmed our results from the Page’s test with an LMM using R version 3.4.2 [53] with tower height as dependent variable, age (z-transformed) and generation as fixed effects and chain number as a random effect using the “lmer” function of the R package lme4 [54]. We tested the overall significance of the model by comparing this full model to a null model lacking generation but comprising age as the covariate and the random effect [55] with an F-test (R function “anova” with the argument test set to “F”).

#### Did children produce a culture-dependent technological product?

The analyses for question 1 would tell us whether there was an increase in tower height along the chains. We also investigated whether this potential increase in performance led to the production of culture-dependent traits, i.e., whether children in the transmission chain condition were able to reach tower heights and shapes which baseline children would not reach independently. For this, we contrasted performance in the transmission chains with baseline performance. We compared tower height in position 10 across all chains and in the last three positions, respectively, with the average height reached in the baseline using a Mann-Whitney U test. We also examined whether children in the transmission chains were ever able to build a tripod.

#### Was there cultural variation between the transmission chains?

We examined whether the chains differed from each other in tower shape, i.e., whether we found the emergence of different “cultural lineages” [7]. For this, we needed comparisons of each transmission chain tower against the remaining nine towers from the same chain (*within-chain similarity*) as well as against all the towers from the remaining seven chains (*between-chain similarity*), resulting in 79 ratings per tower. To get these ratings, we recruited a group of 410 hypotheses-naïve raters on the crowdsourcing platform Crowdflower. The raters were asked to judge the similarity of each tower (both in baseline and the transmission chain, *n* = 101) against all other towers in our dataset using a 7-point scale from *0* (*not similar at all*) to *6* (*very similar*; points in between were not labelled), totaling in 101*100 = 10100 ratings (note that even though raters compared all towers in the dataset against each other, we did not use the data comparing baseline and transmission chain towers for any analysis). This procedure resulted in two independent ratings for each pair of images, which allowed us to calculate the reliability of these judgments [7]. We found a significant correlation between both judgments, *r* = .385, *n* = 5050, *p* < .001. The correlation was low, yet comparable to the one found in Caldwell and Millen’s [7] paper plane task (*r* = .387). As these authors argue, the low correlation coefficient indicates that it was “relatively difficult to objectively judge the similarity of these photographs” (p. 167), especially because such a fine-grained scale was used.

We averaged across the two ratings to calculate the mean similarity of each tower in the transmission chain condition to both 1) the towers within the same chain (n = 9) and 2) the towers of the remaining chains (*n* = 70). An LMM in R using the “lmer” function of the R package lme4 was used to investigate whether towers were more similar to towers within the same chain than to towers from different chains. Mean similarity was used as dependent variable, comparison (within chains/between chains) as a fixed factor, and chain number and generation were entered as random effects. We checked for normal distribution and homoscedasticity of the residuals by visually inspecting a qq-plot and the residuals plotted against the fitted values and found no obvious violation of these assumptions. A Maximum Likelihood test was used to derive the p-value for the predictor “comparison” by comparing the full model including the predictor with a model not including it (using the R function “anova” with the argument “test” set to “Chisq”). To estimate the effect size for the fixed effect, we calculated the marginal R^2^ [56] by using the function “r.squaredGLMM” of the package “MuMIn” [57].

## Results

### Baseline performance

There were no differences in tower height between boys and girls in either condition, so data were collapsed across gender. With regard to age, there was no relationship between age and tower height among baseline children (*r*^2^ = .176, *p* = .446). The lack of an age effect seems to be in contrast to the age effect found in Reindl et al. [17]; however, note that the age range in the baseline condition of the current study (4y5m – 5y1m) was smaller than the age range of the pilot study in Reindl et al. [17] (4y1m – 5y9m), which might have minimized the effect of age on performance differences between children in the current study; in addition, the age effect of the main study in Reindl et al. [17] was only very small (with each SD increase in age (4.31 months), average tower height increased by 2.91 cm (R^2^ = .07)).

In the baseline, children’s constructions had a mean height of 17.20 cm (*SD* = 9.46 cm), ranging from 3 to 32 cm (median = 17.50 cm, mode = 3 cm (*n* = 3)). Children built level-0-, level-1-, and level-2-constructions, with level-1-constructions being the most commonly reached stick height (for definitions see Table 1). The most common tower shapes were *plasticine-only towers* (level-0-construction, *n* = 5) and *hedgehogs* (level-1-construction, *n* = 5). No child built a tripod, supporting the conclusion by Reindl et al. [17] that this construction is likely beyond the inventive power of individual children aged 4 to 6.

### Performance in the transmission chains

There were 80 towers (8 chains with 10 towers) with a mean height of 20.86 cm (*SD* = 11.19 cm), ranging from 1.5 to 59.5 cm (mode (*n* = 14) and median were 17.00 cm). As expected, average tower height in position 1 of the chains (*M* = 23.12 cm, *SD* = 6.67cm) did not differ from the average height reached in the baseline (17.20 cm; independent samples t-test, two-tailed, *t*(27) = 1.617, *p* = .118, Cohen’s *d* = 0.724). Children the transmission chains built level-0-, level-1-, and level-2-constructions (as in the baseline), but also level-3- and level-4-constructions (unlike in the baseline). As in the baseline, level-1-constructions were most commonly built (47.5%), with hedgehogs being the most commonly made shape (*n* = 21). Whereas the maximum height in the baseline was 32 cm (reached by one child), 7 (8.75% of the) children in the transmission chains built towers that were taller than this; these children all built level-3- and level-4-towers, apart from one child who built a level-2-tower.

#### Was there an increase in tower height across generations?

**Fig 1:**
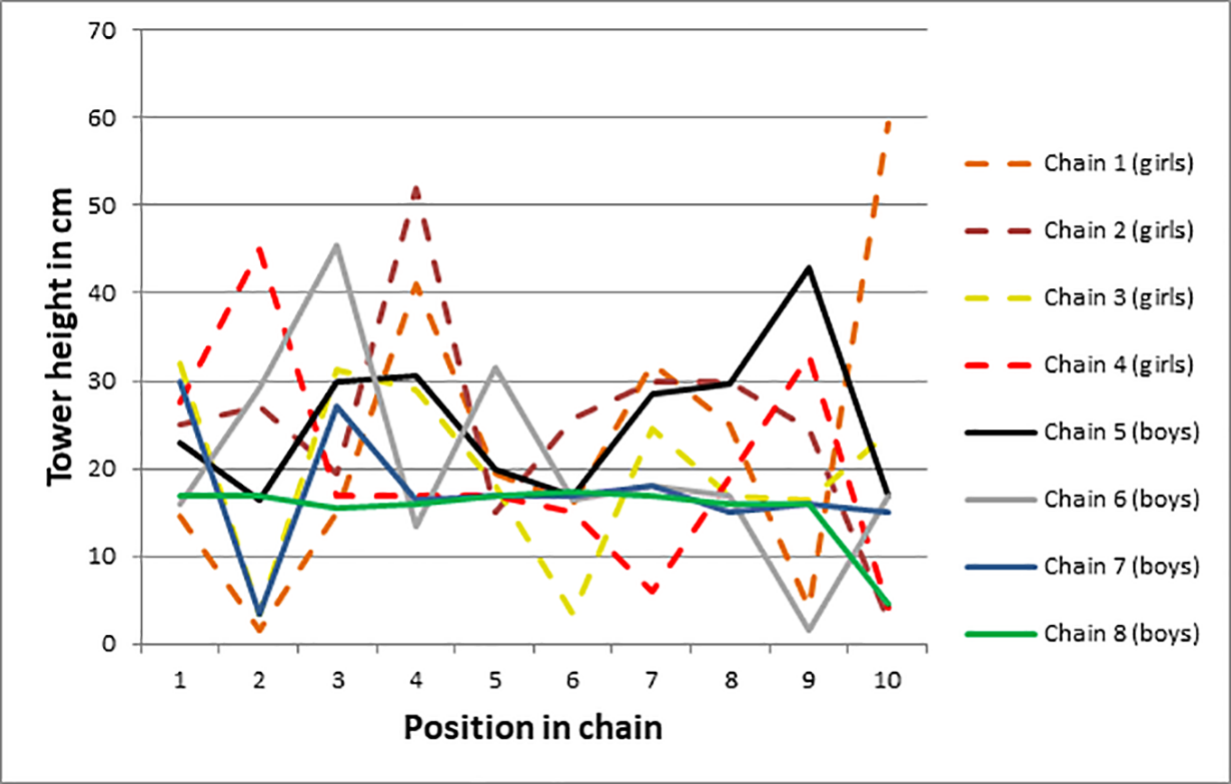
Tower height across the ten positions of the eight transmission chains.

We compared tower height in position 1 (median = 24 cm) to tower height in position 10 (median = 16 cm) across all chains and found no difference in height (Wilcoxon signed rank test, V = 27, *p* = .25). Similarly, there was no difference when tower height across the first three positions (median = 21.25 cm) was compared with tower height across the last three positions (median = 17 cm), Wilcoxon signed rank test, V = 195.5, *p* = .198. In addition, visual inspection of the data suggested that average tower height along the chains did not increase (Fig 1). This was confirmed by the Page’s L test [52]–testing for the presence of a monotonic increase in tower heights across chains–not reaching significance (*L* = 2309.5, *k* = 8, *n* = 10, *p* = .922). Finally, comparing our full LMM against a null model showed that generation did not have an effect on children’s performance., χ^2^(1) = 1.227, *p* = .268.

#### Did children produce a culture-dependent technological product?

Since the previous analyses showed that there was no increase in tower height along the chains, we did not expect children at the end of the transmission chains to have outperformed children in the baseline. We compared tower height in position 10 across the chains (median = 16 cm) with the height in the baseline (median = 17.5 cm) and indeed found no significant difference (Mann-Whitney U test, U = 99.5, *p* = .463). Similarly, there was no difference between tower height in positions 8 to 10 across the chains (median = 17 cm) and tower height in the baseline (median = 17.5 cm), Mann-Whitney U test, U = 252.5, *p* = 1. With regard to tower shape, no child in the transmission chain condition produced a tripod. These results suggest that children in the transmission chains did not produce anything that went beyond what children in the baseline were able to produce independently.

#### Was there cultural variation between the transmission chains?

**Fig 2:**
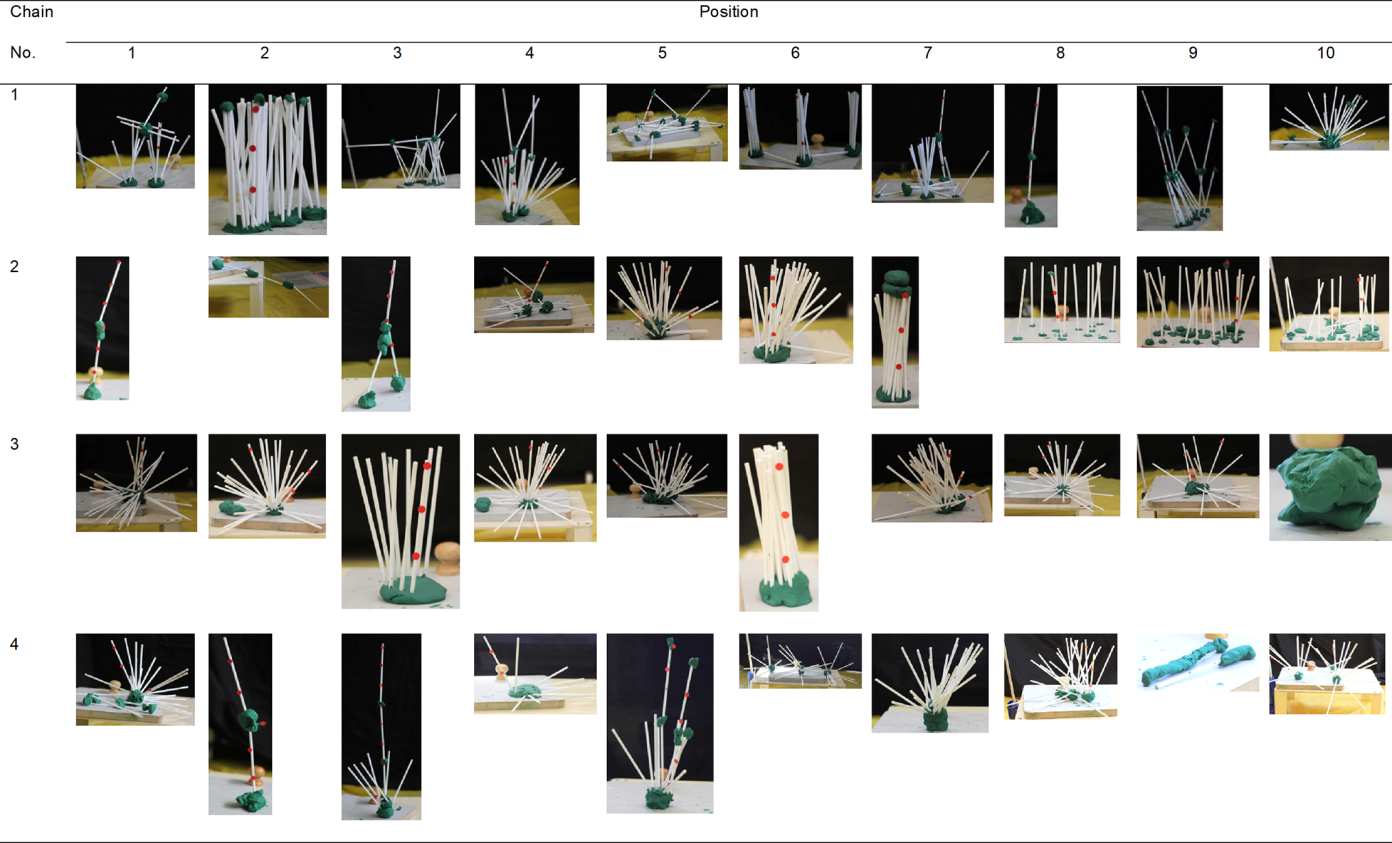
Pictures of towers in the transmission chains (girls). The red sticky dots on the towers were added after the experiment to facilitate telling approximate tower height from the pictures. Starting from the bottom of the construction and going up, we attached one dot at every 5cm.

**Fig 3:**
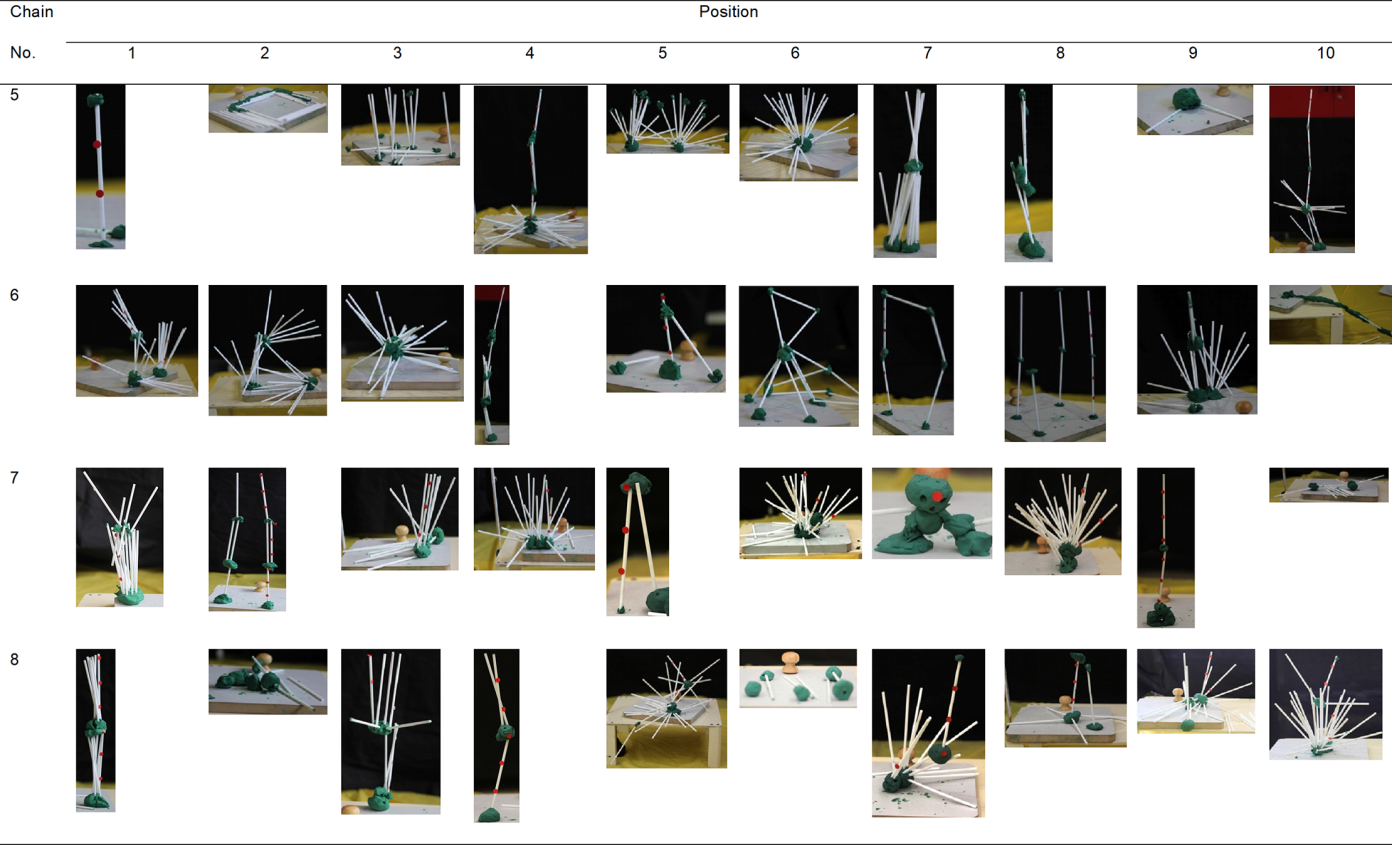
Pictures of towers in the transmission chains (boys).

Mean similarity of the towers within their respective transmission chains was 1.80 (*SD* = 0.73), whereas the mean similarity of the towers to those towers of the remaining chains was 1.54 (*SD* = 0.49). We found that despite similarity ratings for both within- and between chain comparisons being relatively low, the similarity within chains was significantly greater than the similarity between (χ^2^(1) = 7.544, *p* = .006), indicating the presence of chain-specific design traditions. Yet, the effect size for this difference was small: marginal R^2^ = .038. The towers made in the transmission chains are depicted in Figs 2 and 3.

## Discussion

Using the transmission chain paradigm, we investigated whether chains of children between 4.5 and 6 years were able to produce a culture-dependent technological product via the additive ratchet effect in a tower construction task. Children were tested individually and asked to build something as tall as possible. Participants in the transmission chain condition were provided with an endstate demonstration, i.e., they had the opportunity to see the constructions built by the two previously tested children (apart from children in generation 1, who were not presented with any construction, and those in generation 2, who only saw the construction made by the child in generation 1). Children in the transmission chains were found to be slightly older (median = 58 months) than children in the baseline (median = 56 months). However, this is unlikely to alter the interpretation of our findings, as the age difference was not large numerically, and especially because the higher median age in the transmission chains would have likely favoured a positive result: assuming that children’s abilities to build a tower and/or their ability to learn from an endstate demonstration increases with age, one would expect the older children in the transmission chains to outperform the slightly younger children in the baseline. However, even under these circumstances we found the slightly older children in the transmission chains to not perform differently from the baseline children (see results).

We found that tower height did not increase along the chains. Compared to the baseline children, children in the transmission chains did not make towers that went beyond what children in the baseline were able to achieve, with regard to both tower shape and height: in particular, children in the transmission chains did not produce a tripod shape–an efficient solution to the task and regarded to represent cumulative technological design (because of the tower design and absence from baseline). Nor did the average tower height in positions 8 through 10 in the chains differ from the average tower height in the baseline. This is notwithstanding the fact that our experimental manipulation was generally successful in transmitting information: We found evidence for within-chain design traditions, namely in that towers within their chains were more similar to each other than to towers of differing chains. Thus, we detected transmission, but we did not detect the ratchet effect; children in the transmission chains did not create a culture-dependent technological product.

In the introduction, we highlighted the importance of appropriately matching the baseline to the experimental condition. The method we chose in the current study was to match individual children’s testing time across conditions (Method 1, see above). Other studies have used Method 2, in which the total amount of time or the number of generations in the transmission chain condition is applied to the baseline, resulting in baseline individuals to be tested repeatedly [20,43]. While it is so far not clear whether there is only one “correct” way of matching, one could also envision a third method in which individuals in the baseline are given *more* time (individually and overall) since social learning often speeds up the innovative and ratcheting process (see e.g., [39]). Thus, future studies should further improve the matching of asocial control and experimental condition by allocating a greater amount of individual and total testing time to the baseline as compared to the experimental condition.

Despite children’s ability to learn from and copy culture-dependent technological products, even in the absence of action information (see [17], which was also the way the task was presented here in the demonstration condition), children in the current study did not produce a culture-dependent technological product among themselves. This finding contrasts with some studies on adult participants showing that transmission chains of adults working on similarly cognitively transparent tasks (e.g., building paper planes or simple baskets) are able to generate a ratchet effect, even when–similar to our task–participants do not have access to the actions, but only to the end results produced by the previous generations [9,20].

What are possible reasons that we did not find a ratchet effect in our task? One could argue that children’s still developing fine motor abilities might have limited our participants’ ability to create a ratchet effect. While it is true that the task is challenging for young children with regard to its demands on fine motor skills, Reindl et al. [17] have shown that children’s motor skills are already developed enough to create tall and efficient constructions such as the tripod.

As the ratchet effect arguably rests on two key cognitive abilities–high-fidelity transmission and innovation [1,3,5]–we suggest explanations along these two aspects. Regarding transmission, it might be possible that children found it difficult to *recognize good inventions* (i.e., towers that are worth copying), and this might have been due to a still limited understanding of the physical and causal aspects involved in the task. However, we know that children do copy good towers spontaneously [17]. We also know that children can learn from demonstrations even when they do not result in success [51], so one could have expected children to benefit from seeing other children’s towers even if these had not been very tall. In addition, it has been shown that chimpanzees are able to switch from an initial behaviour to another behaviour that yields a higher outcome [58,59], suggesting that the ability to “copy if better” is phylogenetically old and thus could also be expected to be found in young children.

Another possibility is that children would have *required different type(s) of information* in order to learn from these demonstrations (e.g., action information). This is unlikely as it has been shown that children of the same age range as studied here are able to copy towers (including the tripod shape) in this task without additional information–endstate demonstrations (as we used here) proved sufficient to transmit culture-dependent tower shapes [17].

Children could also have lacked the *motivation* to learn from other children’s towers. This might have been because they preferred to make their own construction rather than attending to the demonstrated towers because these towers did not look tall or “spectacular” enough. Alternatively, this might have been because children knew that the demonstrated towers were built by other children rather than (more knowledgeable) adults (here, our instruction said “let me show you what other children did earlier”; in [17], it said “let me show you what I did earlier”). Indeed, studies have shown an age bias in children’s social learning in that children preferentially learn from adults over peers when learning how to operate a puzzlebox, learning novel object labels or simple game rules [60]. However, note that if we had told children that the demonstrated towers were made by adults, children’s very preference to copy adults might have resulted in them copying these towers faithfully while being inhibited to further innovate on top of them. Another reason why children might not have been motivated to learn from the demonstrated towers could have been a lack of a direct incentive to look at these towers or to outcompete them, possibly because children were not explicitly told to make their towers *taller* than the ones shown to them. Nevertheless, it has been shown that children indeed copy a tall tower spontaneously despite there being no direct incentive (apart from the tower being tall) [17]. However, note that in this study children knew that the tripod was built by an adult and this might have been interpreted as a prompt for copying.

Regarding the aspect of innovation, we suggest two reasons for why children in the transmission chains did not show a ratchet effect: First, while children might have been both motivated and cognitively able to learn from the demonstrated towers (assuming no limitation on the transmission aspect), we think it is likely that there was simply “nothing” to be picked up because good innovations occurred too rarely or in too subtle a way. This could be due to children’s lack of innovative skills. Indeed, the literature suggests that children have difficulties innovating, such as when they need to think of making a hook in order to obtain a reward from a tube or when they need to come up with novel strategies to extract even more rewards from a puzzlebox, suggesting that children’s innovative skills are still developing over the pre-school and primary school years [61–65]. Similarly, Hill [35] argued that most innovations are made by adults and children mainly copy and do not innovate themselves as much.

In their review, Carr, Kendal, and Flynn [66] discuss possible prerequisites for making innovations, such as a causal understanding of the task, curiosity, explorative tendencies as well as creativity and divergent thinking (see also [67,68]; but see [69] who found no link between children’s divergent thinking and innovative abilities).With regard to causal understanding, Reindl et al. [17] suggested that 4- to 6-year-old children have a still limited understanding of the physical, causal relations involved in the tower task, which might have made the task–and innovations therein–difficult for them. Similarly, Flynn, Turner, and Giraldeau [70] hypothesized that children might be more likely to innovate on tasks they “believe are easy, or feel expert in” (p.7). However, note that in general, children’s innovative abilities in the material cultural domain are argued to be more pronounced yet than in the domain of social culture (e.g., when learning rituals [71]).

In order to test the hypothesis that children in our study failed to show the ratchet effect because they failed to make initial innovations, future studies could seed transmission chains with evidently good towers and examine what would happen to the seeded trait. If children further improved upon the initial innovation, one could conclude that the lack of a ratchet effect in the current study was probably indeed due to a lack of innovations that were worth learning from. However, if the seeded tower was not improved upon but just transmitted unchanged along the chains or if it disappeared from the chains (subtractive ratchet effect–here with the result of *detriments* to the involved trait), this would show that it is not the lack of a good invention that explains the lack of the ratchet effect; rather, this would point to other cognitive or motivational factors preventing a ratchet effect in children. In addition, such seeded transmission chains will be able to test how salient an improved tower design has to be in order to be adopted by young children.

A second point to consider with regard to the innovation aspect is the possibility that there was no lack of innovations in the first place, but that children were *unable to make “cumulative modifications”* ([72], p.325) on top of them. This could have been due to children’s still developing innovative skills (see above), a motivational issue (as there was no direct incentive for children to make further improvements) or to a feature of our task design: We encouraged children to use the full building time (10 min), to ensure comparable testing times for all children. However, this resulted in several children disassembling their towers to build another one or in their towers falling because children tried to make them even taller as a response to our encouragement. As a result, several children in our study produced as their final tower a tower that did not also represent the tallest tower they made (baseline: 14 out of 21 children (66.7%); transmission chains: 49 out of 80 children (61.3%)). Thus, in the chains, many towers that were presented to the next “generation” were not representatives of the best performance achieved by the previous generation. This might have reduced the opportunities for children to detect the best tower among the demonstrated ones and thus limited the opportunities for a ratchet effect to occur. Therefore, we think that results would likely have looked differently had we passed on each child’s tallest tower to the next generation. Future studies may introduce changes to the study design in order to address this point. For example, the stopping rules could be changed so that trials would end when children consider themselves to be finished rather than when the maximum time is over. Alternatively, one could provide children with several building boards so that they could build several towers within the 10 min timeframe and would be discouraged to disassemble their first tower. However, this would create the challenge of selecting the tower that should be shown to the successor (the tallest one? the one that children prefer?). It could be criticized that our study paradigm (the specific transmission chains we used) made it difficult for innovations to accumulate, for in real life cultural transmission and the ratchet effect rarely stem from a “Telephone Game” type of chain as employed in our study. Cumulative culture typically involves more complex relations. Thus, as Morin (29, p. 125) described, “transmission in real settings can be repeated”, “can come from several distinct individuals, and not just one model”, and a “single diffusion chain can, at any point, branch out into multiple chains”. We agree. Yet, the transmission chain design is rightly widely used as it allows for rigorous testing of social learning mechanisms involved in cultural transmission. For example, our study shows that with our current task design, emulation learning is insufficient to produce a ratchet effect in children, at least when two instances are shown, and in chain lengths of 10. In contrast, more realistic open diffusion studies are often restricted to interpreting correlations, and typically rely less on experimental controls.

Given the literature on children’s poor innovation skills (see above), one might argue that the result of our study–that children failed to create an additive ratchet effect–was foreseeable from the start. We do not think that is the case: As outlined above, the possible reasons for the absence of the ratchet effect in our study are manifold and might not–or not entirely–be explained by children’s lack of innovativeness. In addition, the claim that children have poor innovative skills is still based on a relatively small number of studies and an even smaller number of tasks–more studies are needed before we can conclude that children are “poor innovators” in general. Indeed, a recent study has suggested that children’s innovation skills in the tool-making are affected by the information they are provided with about the functionality of the tool, suggesting that children might be better innovators than currently indicated by the literature [73]. Moreover, future studies could also test slightly older children in order to identify the onset and developmental trajectory of the ability to produce a ratchet effect in human development.

Moreover, it has been suggested that innovation might not be so much a cognitive characteristic of individuals (suggesting the presence of “innovator personalities” or “geniuses”) but that it is rather the result of combining previously isolated pieces of knowledge [74,75]. This combination of information can take place within a single individual who happens to bring together different areas of knowledge, thus becoming a “nexus of previously isolated ideas” ([74], p.4). Yet, with regard to young children it might be unlikely that children become such a nexus given that they have not had much time yet to gather information about the world (and our task in particular). However, the creation of new knowledge can also be brought about by interaction and collaboration of individuals in which new knowledge occurs via co-construction–i.e., in principle even in small experimental settings (*collaborative learning* [33]). Indeed, the study by Dean et al. [12] shows that young children readily work together (i.e., they observe, communicate with, and teach others) to solve novel problems and the study by McGuigan et al. [39] tentatively suggests that groups of children are able to achieve things that are still beyond the scope of individual children. In sum, we believe the question whether children show the additive ratchet effect among themselves–while currently answered to the negative–to be worthy of independent further investigation.

## Supporting information

S1 Table Two methods for matching asocial control conditions to transmission chain conditions. (DOCX)

S2 Table Suggested method for matching an asocial control condition to an open diffusion condition. (DOCX)

S3 Table List of transmission chain studies carried out with children. (DOCX)
