# Supplementary material for: Young children fail to generate an additive ratchet effect in an open-ended construction task
Source: PLoS One. 2018 Jun 18;13(6):e0197828. doi: 10.1371/journal.pone.0197828 (PMC6005566; doi:10.1371/journal.pone.0197828)
Supplement: S1 Table — (DOCX) [file pone.0197828.s001.docx]

S1 Table. Two methods for matching asocial control conditions to transmission chain conditions.

| **Method** | **Time contributed to the final product** | **Transmission chain condition** | **Asocial control condition** |
| --- | --- | --- | --- |
| **Method 1** | Individual contribution: same | Example (chain of 10):  Individual 1 – 10 min  +  Individual 2 – 10 min  +  …  +  Individual 10 – 10 min | Example:  Individual 1: 10 min |
|  |  |  |  |
|  | Total amount: different | 100 min | 10 min |
| **Method 2** | Individual contribution: different | Example (chain of 10):  Individual 1 – 10 min  Individual 2 – 10 min  …  Individual 10 – 10 min | Example:  Individual 1 – 10 min (x10) |
|  |  |  |  |
|  | Total amount: same | 100 min | 100 min |
