# Supplementary material for: Young children fail to generate an additive ratchet effect in an open-ended construction task
Source: PLoS One. 2018 Jun 18;13(6):e0197828. doi: 10.1371/journal.pone.0197828 (PMC6005566; doi:10.1371/journal.pone.0197828)
Supplement: S2 Table — (DOCX) [file pone.0197828.s002.docx]

S2 Table. Suggested method for matching an asocial control condition to an open diffusion condition.

| **Method** | **Time** | **Open diffusion condition** | **Asocial control condition** |
| --- | --- | --- | --- |
| **Method 2** | Individual contribution | Cannot be fixed | Will be determined by total amount |
|  |  |  |  |
|  | Total amount | Can be fixed, e.g., 4h | Can be matched to open diffusion condition: 4h |
