# Supplementary material for: Young children fail to generate an additive ratchet effect in an open-ended construction task
Source: PLoS One. 2018 Jun 18;13(6):e0197828. doi: 10.1371/journal.pone.0197828 (PMC6005566; doi:10.1371/journal.pone.0197828)
Supplement: S3 Table — (DOCX) [file pone.0197828.s003.docx]

Table S3. List of transmission chain studies carried out with children.

| **Type of study** | **Study** | **Participants** | **Conditions** | **Number and length of chains** |
| --- | --- | --- | --- | --- |
| Investigating (additive/  subtractive) ratchet effect | Flynn, 2008 | 80 2- and 3-year-olds | 32 children in 1 no-model control condition  48 children in 4 transmission chain conditions (2 years/opaque, 2 years/transparent, 3 years/opaque, 3 years/transparent) | Total no. of chains: 8  No. of chains per condition: 2  Chain length: 6 |
|  | McGuigan & Graham, 2009 | 64 3- and 5-year-olds | 32 children in 1 no-model control condition  32 children in 4 transmission chain conditions (3 years/opaque, 3 years/transparent, 5 years/opaque, 5 years/transparent) | Total no. of chains: 4  No. of chains per condition: 1  Chain length: 8 |
|  | Tennie et al., 2014 | 80 4-year-olds | 40 children in 1 unseeded transmission chain condition  40 children in 1 seeded transmission chain condition | Total no. of chains: 16  No. of chains per condition: 8  Chain length: 6 |
| Investigating social transmission (without ratcheting aspect) | Horner et al., 2006 | 31 3-year-olds | 15 children in 1 no-model control condition  16 children in 2 transmission chain conditions (lift, slide) | Total no. of chains: 2  No. of chains per condition: 1  Chain length: 8 |
|  | Flynn & Whiten, 2008 | 127 3- and 5-year-olds | 47 children in 1 no-model control condition  80 children in 8 transmission chain conditions (3 years/boys/stab, 3 years/boys/slide, 3 years/girls/stab, 3 years/girls/slide, 5 years/boys/stab, 5 years/boys/slide, 5 years/girls/stab, 5 years/girls/slide, | Total no. of chains: 16  No. of chains per condition: 2  Chain length: 5 |
|  | Hopper et al., 2010 |  | 16 children in 1 no-model control condition  20 children in 1 transmission chain condition | Total no. of chains: 1  No. of chains per condition: 1  Chain length: 20 |
|  | Kempe et al., 2015 | 90 5- to 8-year-olds | 90 children in 3 transmission chain conditions (see 1, seed 2, seed 3) | Total no. of chains: 9  No. of chains per condition: 3  Chain length: 10 |
